# 小细胞肺癌患者治疗前后血清NSE测定的临床意义

**DOI:** 10.3779/j.issn.1009-3419.2011.09.06

**Published:** 2011-09-20

**Authors:** 峰 薛, 莉彦 王, 明岩 张, 莉 蔡

**Affiliations:** 1 150001 哈尔滨，黑龙江省医院肿瘤科 Department of Medical Oncology, Heilongjiang Provincial Hospital, Harbin 150001, China; 2 150040 哈尔滨，哈尔滨医科大学附属第三医院肿瘤科 Department of Medical Oncology, the Third Affiliated Hospital of Harbin Medical University, Harbin 150040, China

**Keywords:** 肺肿瘤, 神经烯醇化酶, 生存, 预后, Lung neoplasms, Neuron specific enolase, Survival, Prognosis

## Abstract

**背景与目的:**

神经烯醇化酶（neuron specific enolase, NSE）是小细胞肺癌（small cell lung cancer, SCLC）敏感的肿瘤标记物之一。本研究旨在研究血清NSE水平作为SCLC诊断和预后因素的实用性。

**方法:**

治疗前后应用电化学发光法测定57例SCLC患者的血清NSE。应用*Kaplan-Meier*法构建生存曲线，采用对数秩检验法（*Log-rank*）进行曲线之间的比较。

**结果:**

SCLC患者治疗前血清NSE水平是患者总生存期的预后因素，广泛期患者与局限期患者的治疗前血清NSE水平有统计学差异（*P* < 0.001），治疗前后NSE的变化率与总生存期无关（*P*=0.084）。

**结论:**

NSE是SCLC患者诊断和评估预后的理想肿瘤标记物。

## Abstract (en)

**Background and objective:**

It has been proven that neuron specific enolase (NSE) was one of the sensitive tumor markers for small cell lung cancer (SCLC). The aim of this study is to investigate the usefulness of serum NSE values as diagnostic and prognostic factors in patients with SCLC.

**Methods:**

Serum NSE was detected by electrochemiluminescence method in 57 SCLC cases pre- and post-treatment. Survival curves were constructed using the *Kaplan-Meier* method and then compared using the *Log-rank* test.

**Results:**

Pre-treatment serum NSE values are prognostic factors for overall survival of patients with SCLC. There was a statistically significant difference in the pretreatment NSE levels of patients who were at an extensive stage compared with the values of those patients who were at a limited stage (*P* < 0.001). The rate of change between the pre- and post-treatment serum NSE levels was not related to the overall survival (*P*=0.084).

**Conclusion:**

NSE is an ideal tumor marker for the diagnosis and assessment prognosis of SCLC.

小细胞肺癌（small cell lung cancer, SCLC）约占肺癌的15%-20%，其与非小细胞肺癌（non-small cell lung cancer, NSCLC）在生物学行为上有着很大的不同，以生长较快、早期转移和对化疗敏感为特点^[1]^。尽管联合化疗有效率可达60%-90%^[2]^，但是预后仍然较差，尤其是已经转移的患者。从20世纪80年代起，研究^[3]^证实了许多可以预测疗效和生存的因素，如性别、疾病分期、一般状态、体重减轻、血红蛋白值、白细胞计数、血小板计数、乳酸脱氢酶（lactate dehydrogenase, LDH）和神经烯醇化酶（neuron specific enolase, NSE）等。

NSE是广泛存在于神经元和神经内分泌细胞的糖酵解酶^[4]^，它是诊断SCLC最敏感的肿瘤标记物之一^[5, 6]^。在初次诊断时，几乎75%的SCLC患者血浆NSE水平升高^[7, 8]^，虽然其为SCLC临床较为普及的肿瘤标记物，但国内外学者对其临床应用也有颇多争议。本研究将针对其临床应用的价值加以研究，并对治疗前后NSE变化率对SCLC患者生存的影响加以讨论。

## 1 资料与方法

### 1.1 患者资料

选取2006年10月-2008年12月在哈尔滨医科大学附属肿瘤医院经病理学诊断（其中2例为肺切除手术病理证实）确诊的SCLC患者57例，年龄29岁-70岁，中位年龄52.5岁。男性39例，女性18例。局限期（limited-stage disease, LD）30例，广泛期（extensive-stage disease, ED）27例。所有患者均接受4个-6个周期的一线化疗。

### 1.2 SCLC的临床分期

根据2011版小细胞肺癌美国国立综合癌症网络（National Comprehensive Cancer Network, NCCN）指南进行临床分期：局限期为病变局限于同侧胸腔，可被包含在一可耐受的放射治疗野内；广泛期为病变超出同侧胸腔，包括恶性胸腔及心包积液和血性转移。

### 1.3 治疗方案

57例患者均采用EP（或CE）方案化疗，具体方案：依托泊苷（100 mg/m^2^, d1-d3）联合顺铂（总量75 mg/m^2^，d1-d3）或卡铂（AUC=6, d1），3周重复。其中，26例接受了胸部放射治疗（总量46 Gy-50 Gy），1例行全脑预防性照射（prophylactic cranial irradiation, PCI）。

### 1.4 随访

一线治疗后，每3个月-4个月对所有患者行胸部CT、上腹部彩超、血清NSE，必要时行上腹部CT、头部CT或MRI检查。记录患者疾病进展时间（time to progression, TTP）及复发转移时血清NSE水平，直至死亡。

### 1.5 NSE水平测定

所有患者在诊断时、每周期化疗前，均留取血标本（避免溶血），1 h内送至检验部门，离心并分离血清，应用Roche全自动电化学发光免疫分析仪（Elecsys2010）及Roche神经元特异性烯醇化酶定量测定试剂盒（电化学发光法）对样本血清进行测定。参照分析仪及试剂盒厂商评定标准（Elecsys NSE Multicenter Evaluation; study no.B99P005, 7/2001），根据患者群体的参考范围变异性制订NSE参考值范围（0 ng/mL-15.2 ng/mL）。

### 1.6 统计学方法

采用SPSS 13.0进行数据分析。对同生存时间可能存在关联的因素，采用*Kaplan-Meier*进行单因素分析，采用*Log-rank*法进行检验。多因素生存分析采用*Cox*比例风险模型。用*t*检验和线性回归方法对NSE水平的影响因素进行分析。以*P* < 0.05为差异有统计学意义。

## 2 结果

### 2.1 患者初诊NSE水平与生存时间的关系

患者初诊NSE水平的均值为42.40 ng/mL，据此将患者分为NSE高于均数和低于均数的两组，根据*Kaplan-Meier*方法估算两组的中位生存时间，并绘制生存曲线（图 1）。

**1:**
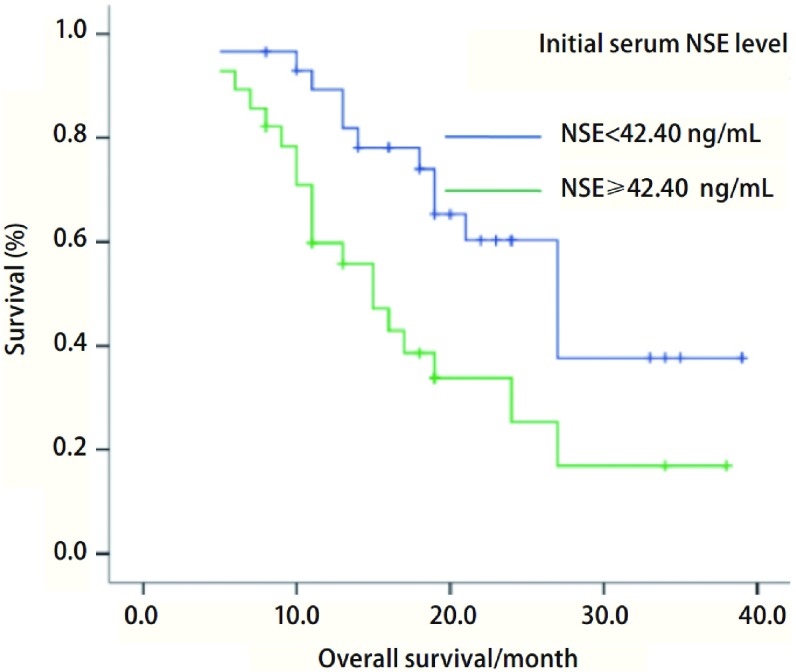
患者初诊神经烯醇化酶水平与生存的关系（*P*=0.011） The relationship between initial neuron specific enolase (NSE) and overall survial(*P*=0.011)

NSE < 42.40 ng/mL组患者（*n*=29）的中位生存时间为27个月（95%CI: 20.726-33.274），NSE≥42.40 ng/mL组患者（*n*=28）的中位生存时间为15个月(95%CI: 10.500-19.500），两组患者的生存时间存在统计学差异（*P*=0.011）。

从图 1中可以看出，高NSE和低NSE组患者生存曲线明显分离，低NSE（NSE < 42.40 ng/mL）组患者的累积生存概率始终高于高NSE组患者。根据*Cox*比例风险模型估算，初诊NSE对应的风险比为1.027（*P*=0.005, 95%CI: 1.009-1.029），即初诊（治疗前）NSE水平每升高一个单位，患者的死亡概率平均增加约2.7%。

### 2.2 临床分期与初诊患者的NSE水平之间的关系

LD患者（*n*=30）的NSE为（34.04±29.41）ng/mL，ED患者（*n*=27）的NSE为（94.31± 65.27）ng/mL，两组比较有统计学差异（*t*=4.571, *P* < 0.001）。

### 2.3 患者初诊NSE水平与TTP之间的关系

患者初诊NSE水平与TTP之间无相关性（*r*=0.223, *P*=0.306）。

### 2.4 患者治疗前后NSE的变化率与生存之间的关系

为了衡量在治疗过程中NSE水平的变化是否对患者的预后产生影响，定义NSE变化率=（初诊NSE-二次化疗后NSE）/初诊NSE，结果用百分率表示。将患者分为NSE变化率 < 50%和≥50%两组，绘制生存曲线（图 2），并用*Log-rank*检验比较两组之间的差异。结果表明，NSE变化率 < 50%组患者（*n*=13）的平均生存时间为28.556个月（95%CI: 21.599-35.514），NSE变化率≥50%组患者（32例）的平均生存时间为20.529个月（95%CI: 16.376-24.682），两组间无统计学差异（*P*=0.084）。

**2:**
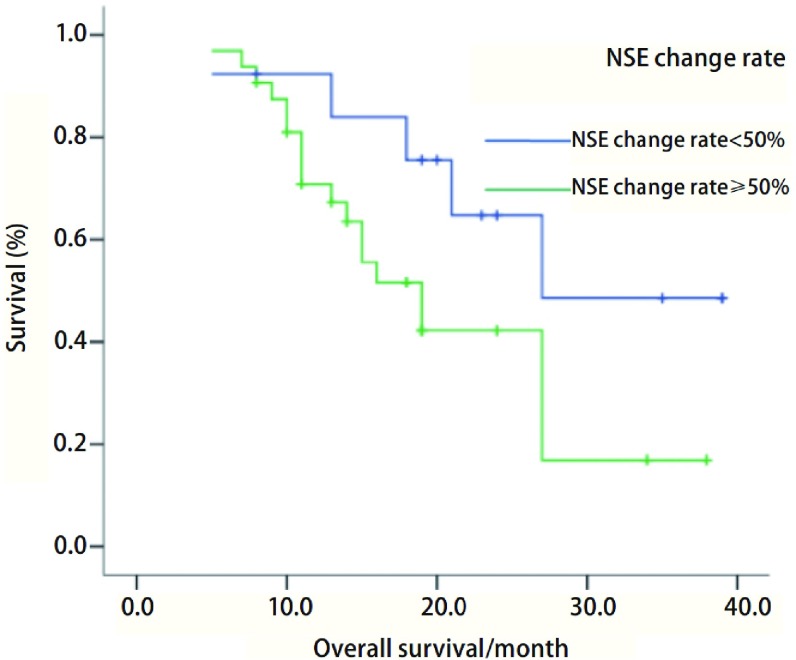
患者NSE变化率同患者生存的关系（*P*=0.084） The relationship between rate of change of NSE and overall surviva (*P*=0.084)

从图 2可见，NSE变化率低组和高组患者之间的生存曲线有交叉，但在患病后期NSE变化率低的患者生存概率高于NSE变化率高的患者。采用*Cox*比例风险模型推算NSE变化率对患者生存时间的影响，结果表明NSE变化率对患者生存时间的影响不明显（*P*=0.058）。

## 3 讨论

在临床实践中，理想的肿瘤标记物应该可以筛选和早期诊断疾病、评估疗效、判断预后并且在治疗和随访中可以监控病情变化。Ebert^[9]^认为血清NSE测定是诊断SCLC的首选肿瘤标志物。另有研究^[10-12]^表明，在SCLC患者中，有45%-50%的LD患者及85%-98%的ED患者血清NSE升高，已成为影像学诊断的重要补充手段。

血清NSE的测定对于SCLC的诊断及鉴别诊断有重要的临床意义。但有7%-42%的NSCLC患者和11%-14%的非恶性肿瘤患者，NSE升高呈假阳性^[13]^。而血清NSE水平低于临界值的一部分SCLC患者，经病理证实为含有混合性肿瘤细胞，可能与神经内分泌细胞含量偏低及血中释放少有关。有研究^[14]^报道NSE鉴别SCLC与NSCLC及肺部良性肿物的敏感性为43%。

一般来说，治疗前患者的NSE水平与SCLC患者分期有一定相关性。Pinson^[15]^也曾指出ED患者的血清NSE平均水平要远高于LD患者。Molina等^[16]^研究结果表明，NSE在LD-SCLC敏感性较低，正常人和LD-SCLC患者的NSE水平差异性小。在我们的研究中，LD与ED的NSE水平具有统计学差异（*P* < 0.001）。在初诊判断SCLC分期时，对于存在隐匿转移灶的ED患者，NSE水平可能为其提供有价值的线索。但暂无文献报道血清NSE水平与肿瘤负荷相关。

血清NSE水平对生存期具有预后价值。Jorgensen等^[17]^选取了来自9个治疗中心的770例患者，经过统计分析认为，治疗前血清的NSE水平是影响患者生存期最重要的预后因素，其次是患者的一般状态和疾病分期。在我们的研究中，应用NSE平均值将患者分组，两组的中位生存时间相差12个月，表明初诊NSE较低者有较长的生存时间。初诊NSE水平每升高一个单位，患者的死亡概率平均增加约2.7%。因入组病例数的关系，对于NSE < 15.2 ng/mL与15.2 ng/mL-42.4 ng/mL两组患者的总生存时间未做比较。

有研究^[15]^表明SCLC复发患者的血清NSE，在复发病灶被探测4周-12周之前就明显升高，但治疗前后NSE的水平对于判定SCLC复发并无意义^[18, 19]^。Van de Pol等^[7]^研究发现，随访观察NSE的浓度变化与肿瘤复发转移有关，但与转移的部位无关。在本研究中，有18例患者在影像学证实疾病进展后测定了NSE，其中17例（94%）患者NSE升高，且17例中4例初诊时NSE水平正常，13例于治疗后NSE下降，而疾病进展后NSE又升高，这些特点支持随访NSE浓度变化与疾病进展相关的结论。

有报道^[18]^表明，治疗反应较好者，其治疗前NSE水平较低，治疗后NSE水平与化疗疗效无相关性，治疗前和治疗后NSE水平的变化与治疗疗效、无疾病进展生存和总生存期无明显相关性。在本研究中，我们统计治疗前后NSE变化率与生存时间的关系，得到了相似的结果，即无统计学差异（*P*=0.084）。另外，初诊时NSE的值与TTP之间也无相关性（*P*=0.306）。

综上所述，NSE是SCLC患者诊断及鉴别诊断的理想标记物，NSE还可用于评估患者的预后，并对判断疾病进展有一定的预测意义。但化疗后其变化率与患者的生存无相关性，故治疗开始后，随访NSE水平对患者的实际临床意义究竟多大？有待进一步研究探讨。
